# Ecotoxicity of oil sludges and residuals from their washing with surfactants: soil dehydrogenase and ryegrass germination tests

**DOI:** 10.1007/s11356-020-11300-2

**Published:** 2020-11-11

**Authors:** Diego Ramirez, Liz J. Shaw, Chris D. Collins

**Affiliations:** Department of Geography and Environmental Science, University of Reading, Reading, RG6 6DW UK

**Keywords:** Oil sludge washing (OSW), Surfactants, Toxicity, Dehydrogenase activity (DHA), Germination test, Ryegrass

## Abstract

Oil sludge washing (OSW) with surfactants and co-solvents is used to recover the oil, and this process leaves some residuals (sediments and surfactant solution). Currently, there are no data on the ecotoxicological effects of these OSW residuals from different sludges. This study evaluated the toxicity of OSW residuals from washing four types of oil sludges with five surfactants (Triton X-100 and X-114, Tween 80, sodium dodecyl sulphate (SDS) and rhamnolipid) and a co-solvent (cyclohexane). The toxicity of the residuals was evaluated with the impact on the soil microbial dehydrogenase activity (DHA) and ryegrass (*Lolium perenne*) seed germination. There was a high DHA detected directly in the sludges and all OSW residual combinations, but this activity could not be attributed to the DHA itself but to some chemical interferences. The DHA was then tested in the soils amended with the OSW residuals to simulate a bioremediation scenario. There were no chemical interferences in this case. In general, the INTF concentrations were significantly higher at low concentrations, 1 and 5% (*p* < 0.01). There were no significant differences in the DHA at high concentrations of OSW residuals (10, 25 and 50%) which implied that the concentration of the contaminants is not directly proportional to the levels of ecotoxicity. Unexpectedly, the INTF values of the 10, 25 and 50% rhamnolipid-OSW residuals were significantly lower than the Triton X-100 residuals. The ryegrass germination rates were higher than 70% with no apparent phytotoxicity symptoms in the seedlings. Particularly, there was a highly significant negative effect of the residuals on the germination rates at high concentrations (*p* < 0.01). Given that the extractable petroleum hydrocarbon (EPH) concentrations in the OSW residual-amended soils in both DHA and germination tests were very low (13–21 ppm), other co-contaminants could be contributing to the toxicity. These findings implied that biotreatment techniques can be applied to treat the OSW residuals if necessary.

**Supplementary Information:**

The online version contains supplementary material available at 10.1007/s11356-020-11300-2.

## Introduction

Approximately one billion tonnes of oil sludges have been accumulated worldwide (Mirghaffari 2017), and this substantially affects the functioning of the petroleum industry and impacts the environment. These wastes are principally composed of crude oil, water and sediments. Moreover, oil sludges can have some traces of heavy metals such as copper (Cu), lead (Pb), vanadium (V) and chromium (Cr) (Hu et al. 2013). The strategies to treat these wastes are based on contaminant reduction or oil recovery techniques. Following the current waste management trends that indicate the importance of waste reduction, reuse and recycle (Sakai et al. 2011; European Parliament 2008), the treatment of oil sludges has been recently aimed at oil recovery techniques (Gumerov et al. 2017; Nezhdbahadori et al. 2018). Therefore, oil sludge washing (OSW) with surfactants has been applied to treat the oil sludges as an oil recovery method (Zhang et al. 2012; Liang et al. 2017; Duan et al. 2018; Liu et al. 2018a), and co-solvents can be added to enhance the extraction of the oil (Hwa et al. 2016; Kuppusamy et al. 2017). Surfactants can reduce the interfacial tension of the W/O macroemulsion in the sludge. Then, the agitation during the washing breaks the emulsion (Rosen and Kunjappu 2012).

Since the main aim of the OSW is the oil recovery, much less is known about the residuals left by this process. The OSW residuals are composed by the surfactant solution with sediment and water from the oil sludge. These residuals can also have some oil and heavy metal residues (Hu et al. 2013). If the OSW residuals still have oil and heavy metal remnants, it is necessary to evaluate the ecotoxicity to determine if bio-remedial methods such as landfarming and phytoremediation are suitable as further treatments. It is relevant to assess the ecotoxicity of these residuals because these tests are sensitive to the bioavailable fraction of the contaminant. Therefore, these tests should be considered as a complement to other chemical analyses (Wilke et al. 2008; Onwosi et al. 2019) such as the determination of oil, heavy metals and extractable petroleum hydrocarbons (EPH) concentrations.

Due to the high amount of oil sludges and petroleum-related waste produced in the industry, the waste treatment companies need to perform simple-to-use protocols for the rapid assessment of the composition of oil sludges (Jin et al. 2013; Ramirez et al. 2019) to decide which treatments these wastes should follow (e.g. oil recovery and/or contaminant reduction methods). Following this idea of seeking simple-to-use methods, it is relevant as well to find rapid ecotoxicity tests to assess any effect of the bioavailable part of the contaminant in the soil microbiota or plants if a biotreatment (e.g. bioremediation or phytoremediation) is the alternative chosen to remediate the residuals or oil sludges. Even though methods such as earthworm (*Eisenia foetida*) mortality, *Daphnia magna* immobilization and luminescence inhibition with *Vibrio fischeri* (Alvarenga et al. 2007; Alvarenga et al. 2016) give a good assessment of the toxicity of other types of organisms, these are time-consuming, need an extra preparation of material and adequation of the model organisms and are not pertinent in the present biotreatment scenario.

In the case of assessing the toxicity in microorganisms for an eventual bioremediation process, microbial enzyme assays can be considered. These assays are frequently available and simple-to-use methods (Małachowska-Jutsz and Matyja 2019). The dehydrogenase activity (DHA) test measures the soil aerobic microbial oxidation using the water-soluble iodonitrotetrazolium chloride (INT) as an artificial electron acceptor (Shaw and Burns 2006). The dehydrogenases are known to be one of the most essential soil enzymes (Wolf et al. 2020). These enzymes are a type of oxidoreductases used by the soil microbiota that are susceptible to petroleum hydrocarbons, so the DHA test can be used as a toxicity marker for the soil microorganisms (Suleimanov et al. 2005; Kaczyńska et al. 2015). Moreover, Wei et al. (2019) reported that the DHA was positively correlated with the total petroleum hydrocarbon (TPH) degradation rate. Besides, Onwosi et al. (2019) mentioned that the DHA, along with other enzymatic assessments such as the catalase and lipase tests, were the most bio-responsive parameters in an oil-contaminated soil. Consequently, the DHA is a complete representation of the soil microbiota activities because it shows the status of most of the biochemical reactions performed by the microorganisms (Małachowska-Jutsz and Matyja 2019; Mansour et al. 2019). The DHA test is considered to be sensitive to any physicochemical changes that affect the soil microbiota, so it can elucidate the effects in the soil health (Campos et al. 2019).

If a phytoremediation scenario is considered where the OSW residuals are amended to the soil, plant species such as the ryegrass (*Lolium perenne*) can be used. This plant is tolerant to petroleum hydrocarbons (Olson et al. 2003; Kaimi et al. 2006; Barrutia et al. 2011), so it has been used in phytoremediation studies, particularly in crude oil-contaminated soils (Kirk et al. 2005; Tang et al. 2010; Cook and Hesterberg 2013; Huang et al. 2019; Wei et al. 2019) and oil sludge-contaminated soils (Muratova et al. 2008; Wang et al. 2016). Ryegrass is commonly used in seed germination toxicity tests (Lin and Xing 2007), and it has been used in germination tests of oil-contaminated soils (Wei et al. 2019). Therefore, this ecotoxicity test can be performed to evaluate the potential use of a plant in a phytoremediation process of an oil-contaminated matrix (Banks and Schultz 2005; Wang et al. 2019).

This study aimed to perform toxicity tests of different types of oil sludges and their OSW residuals in soil microorganisms with the DHA test and in plants with the germination of ryegrass for assessing if these residuals are suitable for further biotreatment processes (i.e. bioremediation and/or phytoremediation). To our knowledge, this is the first study that uses soil microbiota dehydrogenase test to assess the toxicity of oil sludges and the OSW residuals from different oil sludges.

## Materials and methods

### Characteristics of the oil sludges

Four oil sludges from different sources obtained in the UK were used in this study. An oil drilling sludge (ODS), two waste engine oil sludges from two processes to remove metals by gravitational settling (STS) and centrifugation (RS) and an oil refinery sludge (NSC). All oil sludges had semi-solid consistencies at room temperature. Table S1 shows the physicochemical characteristics such as dry and wet contents and extractable petroleum hydrocarbons (EPH) of the sludges. Table S2 shows the concentrations of trace elements, including the heavy metals, in the sludges. These analyses were done in a previous study (Ramirez et al. 2019).

### Surfactants and co-solvent

Five surfactants were used. One biosurfactant, 90% pure rhamnolipid obtained from *Pseudomonas aeruginosa* (AGAE Technologies. Corvallis, Oregon, USA), and four synthetic surfactants, the anionic sodium dodecyl sulphate, SDS (BDH Laboratory supplies) and the non-ionic Tween 80, Triton X-114 and Triton X-100 (Sigma-Aldrich). The stock surfactants solutions were dissolved in ultrapure water (18.2 MΩ/cm). The concentrations of the stock solutions were 10% (*w*/*v*) for rhamnolipid and SDS and 10% (*v*/*v*) for Triton X-100, Triton X-114 and Tween 80. These surfactants have been used before in oil recovery and washing studies. Cationic surfactants were not included in this study because these surfactants tend to be adsorbed onto the sludge particles which are generally negatively charged. Therefore, this type of surfactants is not suitable for oil recovery purposes (Wesson and Harwell 2000). Moreover, cationic surfactants are less benign to the environment than other types of surfactants.

The co-solvent used was cyclohexane (high-purity, HPLC grade, Fisher Scientific). Cyclohexane was selected in a previous oil recovery study from OSW processes (Ramirez et al. 2020). It was found that the oil recovery rates were not significantly different between cyclohexane and one of the most commonly used solvents, toluene. Moreover, cyclohexane was preferred because it is less hazardous to the environment compared with toluene.

### Oil sludge washing

The residuals were obtained at bench scale from the following OSW process. Briefly, the oil sludge (5 g), surfactant [5:1 ratio to oil sludge and at 5CMC, critical micelle concentration; the highest ratio and concentration used in previous oil recovery studies with these oil sludges samples (Ramirez and Collins 2018; Ramirez et al. 2020)], and cyclohexane (1:1 ratio to oil sludge) were added to a 40-ml vial. The vial was then agitated at 250 rpm for 60 min and left for 12 h to settle. Three layers were observed as follows: oil and co-solvent (top), water and surfactant (middle) and sediments (bottom). The middle and bottom layers were taken and used as OSW residuals (Ramirez and Collins 2018).

### Toxicity tests of the OSW residuals

#### Sample preparation

Table 1 shows the varying concentrations of OSW residuals amended to the soil that were used in the toxicity tests (DHA and germination test).

**Table 1:** Concentrations of oil sludge washing (OSW) residuals amended to soil used in the toxicity tests

Toxicity tests	OSW residuals (%) amended to soil				
	1%	5%	10%	25%	50%
Dehydrogenase activity	All sludges (Triton X-100, SDS)	All sludges (Triton X-100, SDS)	STS (Triton X-100, Rhamnolipid, SDS)	STS (Triton X-100, Rhamnolipid, SDS)	STS (Triton X-100, Rhamnolipid, SDS)
Seed germination	ODS, STS (Triton X-100)	ODS, STS (Triton X-100)	ODS, STS (Triton X-100)		

*ODS* oil drilling sludge, *STS* waste engine oil sludge with a metal removal pre-treatment by gravitational settling, *SDS* sodium dodecyl sulphate

First, the sludges and OSW residuals were analysed with the DHA test to have a comprehensive evaluation of the toxicity and to assess the incubation time in this test. All surfactants were added at 5CMC which was the highest concentration used in previous oil recovery studies with these sludges (Ramirez and Collins 2018; Ramirez et al. 2020). OSW residual concentrations in soil were taken from Singh and Agrawal (2007) and Mazen et al. (2010). The selection of the sludges and surfactants in the OSW residuals was based on the results of the preliminary DHA test which evaluated the OSW residuals in the entire range of combinations of sludges and surfactants (see “Determination of the toxicity of OSW residuals in the soil microbiota” for further clarification).

Soil (20-cm depth) used in the toxicity tests was taken from Sonning, Berkshire, UK (SU 762754; GB grid). Fresh field moist soil was sieved (2.0 mm) and kept at 4 °C for the DHA test, whereas for the germination test, the soil was dried at 40 °C for 4 days, further sieved (2.0 mm) and ground (Greene et al. 1989). The OSW residuals were then blended with the processed soil by mechanical mixing (60 rpm) with a Stuart roller mixer SRT9D (Bibby Scientific Ltd.) for 60 min. Each type of OSW residual obtained was finally separated into subsamples for the toxicity tests.

The DHA and ryegrass germination tests (soil-based assays) were selected as the toxicity assessments following a possible scenario of further treatment of the OSW residuals with landfarming and phytoremediation.

#### Dehydrogenase activity test

The method used was adapted by Shaw and Burns (2006). The lab material was autoclaved. The iodonitrotetrazolium chloride (INT), supplied by Manchester Organics (UK), was previously dissolved in ultrapure water (18.2 Ω/m) for 4 h with constant agitation, and it was further sterilized by adding the solution with a syringe through a 0.2-μm filter. One (1) gram of sample was added to a sterile McCartney bottle (28 ml) with a 4 ml of 0.2% (*v*/*w*) INT solution. The vial was then left in the dark at 25 °C, and the incubation time (24 h) was determined by a preliminary DHA test (see “Determination of the toxicity of OSW residuals in the soil microbiota”). Next, the extractant solution (10 ml of N,N-dimethyl formamide/ethanol; 1:1, *v*/*v*) was added to the sample, and it was constantly agitated (200 rpm) at 25 °C in a dark room for 60 min. The combination of sample and extractant (2 ml) was then centrifuged at 11,600×*g* for 5 min. The absorbance of the supernatant was finally analysed at 464 nm (Cecil Digital Ultraviolet Spectrophotometer, Series 2, CE 292). The reduction of the water-soluble INT was detected by the conversion of the sample colour from yellow to purple, which showed the reduced water-insoluble compound, iodonitrotetrazolium formazan (INTF) (Shaw and Burns 2006). A range of concentrations from 0 to 25 μg ml^−1^ of INTF working standard (Sigma-Aldrich) dissolved in 5:2 (*v*/*v*) extractant and ultrapure water (18.2 Ω/m) were used for the calibration curve.

A biotic control (sample with water) and abiotic control (sample with no incubation time because the OSW residuals and sludge could not be sterilized) were used to correct the absorbance of the samples. The real INTF concentration was calculated using the equation from the calibration curve and by multiplying the total volume of INT and extractant used (i.e. 14 ml). A two-way analysis of variance was used to assess the effect of surfactant type and OSW residuals in the DHA, and a further post hoc Tukey’s test (*α* = 0.05) was also done. The statistical analyses were done using Minitab 17.3.1 (Minitab Inc.), and the graphs were done with GraphPad Prism 7.01 (GraphPad Software, Inc.).

#### Ryegrass seed germination toxicity test

The test was based on Greene et al. (1989) and a standard guide (ASTM-E1963-09 2014). Ryegrass (*Lolium perenne*) seeds were supplied by Emorsgate Seeds (UK). The contaminated soil (100 g) was added to 15 cm × 1.5-cm plastic Petri dishes (Sigma-Aldrich). Seeds of equal size were selected and sowed (*n* = 25; 5 columns × 5 rows). The water-holding capacity was adjusted with ultrapure water (18.2 MΩ/cm) to 85%. The soil was covered with sand (90 g), and the Petri dish was closed with a lid. Petri dishes were exposed to a daily cycle of 13 h of light and 11 h of dark, and these were opened for aeration daily. The germination rate (%) was calculated as the number of emerging seedlings over the total number of sowed seeds (Besalatpour et al. 2008). The relative humidity and temperature values were registered every hour with an electronic data logger (RH and Temp Datalogger v. 1.5). The germination controls used for this test included a surfactant solution in soil (5% TX100; see “Determination of the toxicity of OSW residuals in the soil microbiota” for an explanation about this choice), a positive (Kettering loam soil), and two negative controls (diesel and boric acid, H_3_BO_3_). Differences between the mean germination rates of OSW residual-treated and non-treated soils were analysed using a paired *t* test (*α* = 0.05). The statistical analyses were done using Minitab 17.3.1 (Minitab Inc.).

The pH of the soil was measured before and after the germination toxicity test. Briefly, the sample (10 g) was added into a 50-ml centrifuge tube with 25 ml of ultrapure water (18.2 MΩ/cm). The sample was thoroughly mixed at 30 rpm using an end-over-end shaker for 15 min (MAFF 1986). The pH value was assessed with a FiveEasy™ pH meter (Mettler Toledo).

## Results and discussion

### Determination of the toxicity of OSW residuals in the soil microbiota

The toxicity in the soil microbiota of the OSW residuals from the washing of oil sludges from different sources was determined by the DHA test. Regarding the characteristics of the oil sludges used in this study, Table S1 shows that ODS had the highest dry matter content value (87% ± 0.02) due to the high presence of solids in the mud obtained during the oil drilling works. Consequently, it had the lowest water content (13% ± 0.02). Also, this sludge had the highest C_10_-C_18_ aliphatic fraction (98%). Although RS and STS came from the same source, the solid content was significantly higher in the former than the latter (*p* < 0.01). Nevertheless, the organic material content was not significantly different (*p* = 0.104). Both RS and STS had the highest percentage of the heavy C_19_-C_36_ aliphatic fraction with 83 and 85%, respectively (Table S1). NSC had the lowest solid content among the sludges (1% ± 0.07) due to the high dried organic content (39% ± 2). This high organic material content in NSC could be related to its high total EPH concentration (68,000 ± 6070). The metal concentrations in these sludges (Table S2) were under the limit of landfilling-acceptable metal concentrations established by the European Union (Kriipsalu et al. 2008), except for Zn which can be found in petroleum-associated porphyrin compounds (Jasmine and Mukherji 2015). All sludges had high concentrations of Ca and Fe (> 1000 μg g^−1^).

**Fig. 1:**
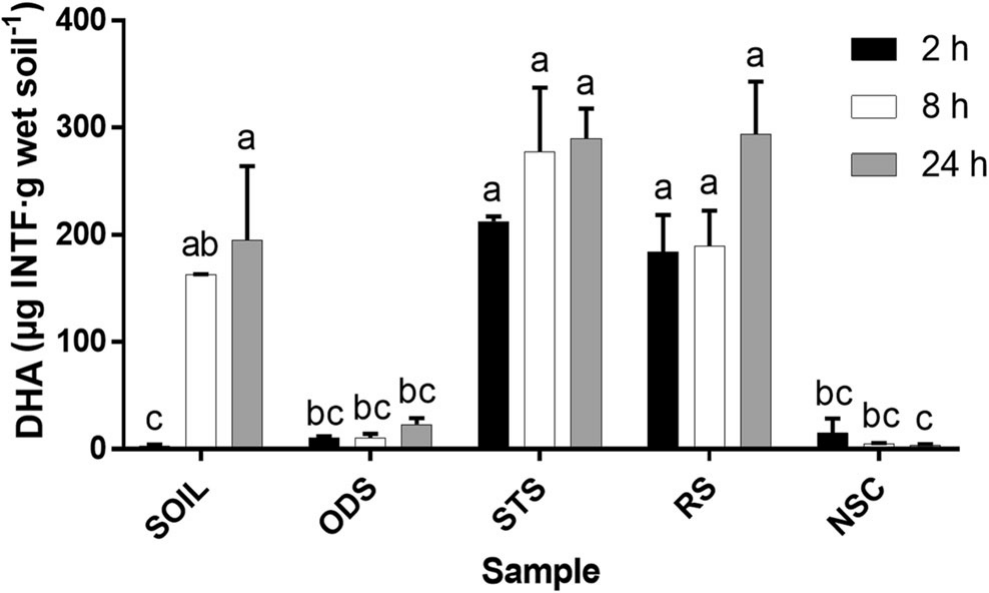
Dehydrogenase activity (DHA) in soil and oil sludges at different incubation times. A Tukey’s test compared the samples (soil and oil sludges) with incubation times. Values with the same letters are not significantly different (*p* > 0.05). The bars indicate the standard error of the mean, SEM (*n* = 3). ODS: oil drilling sludge. STS: waste engine oil sludge with a metal removal pre-treatment by gravitational settling. RS: waste engine oil sludge with a metal removal pre-treatment by centrifugation. NSC: oil refinery sludge. INTF: iodonitrotetrazolium formazan

Figure 1 shows the production of INTF due to the activity of the dehydrogenase in the sludges (ODS, STS, RS and NSC) and soil at different incubation times.

The interaction of the incubation time and sludge type was highly significant (p < 0.01). There were highly significant effects on the incubation time and the sample (soil and sludges) factors (p < 0.01). The soil DHA values had a significant increase in incubation times (*p* = 0.002). In addition, the DHA had a non-significant increase with the incubation time in the ODS, STS, and RS samples; the DHA was detectable at 24 h. In general, any DHA detected in the oil sludges and OSW residuals revealed that the oil hydrocarbons present could be a carbon source for the microbial hydrocarbon degraders (Serrano et al. 2008). The DHA in STS and RS at 2 h was significantly higher than soil, ODS and NSC (*p* < 0.01). These apparently higher DHA values in the STS and RS samples compared with the soil, ODS and NSC samples indicated spurious activity due to chemical reactions unrelated to the activity of the dehydrogenase and which were undetected by the abiotic controls. Although the DHA in the NSC sludge did not present a significant decrease with time (*p* = 0.449), the DHA values at 8 h and 24 h were significantly higher (*p* = 0.004 and *p* = 0.026, respectively) compared with the DHA value at 0 h, except for the value at 2 h (*p* = 0.186). Consequently, it can be inferred that NSC had detectable DHA values at 24 h.

**Fig. 2:**
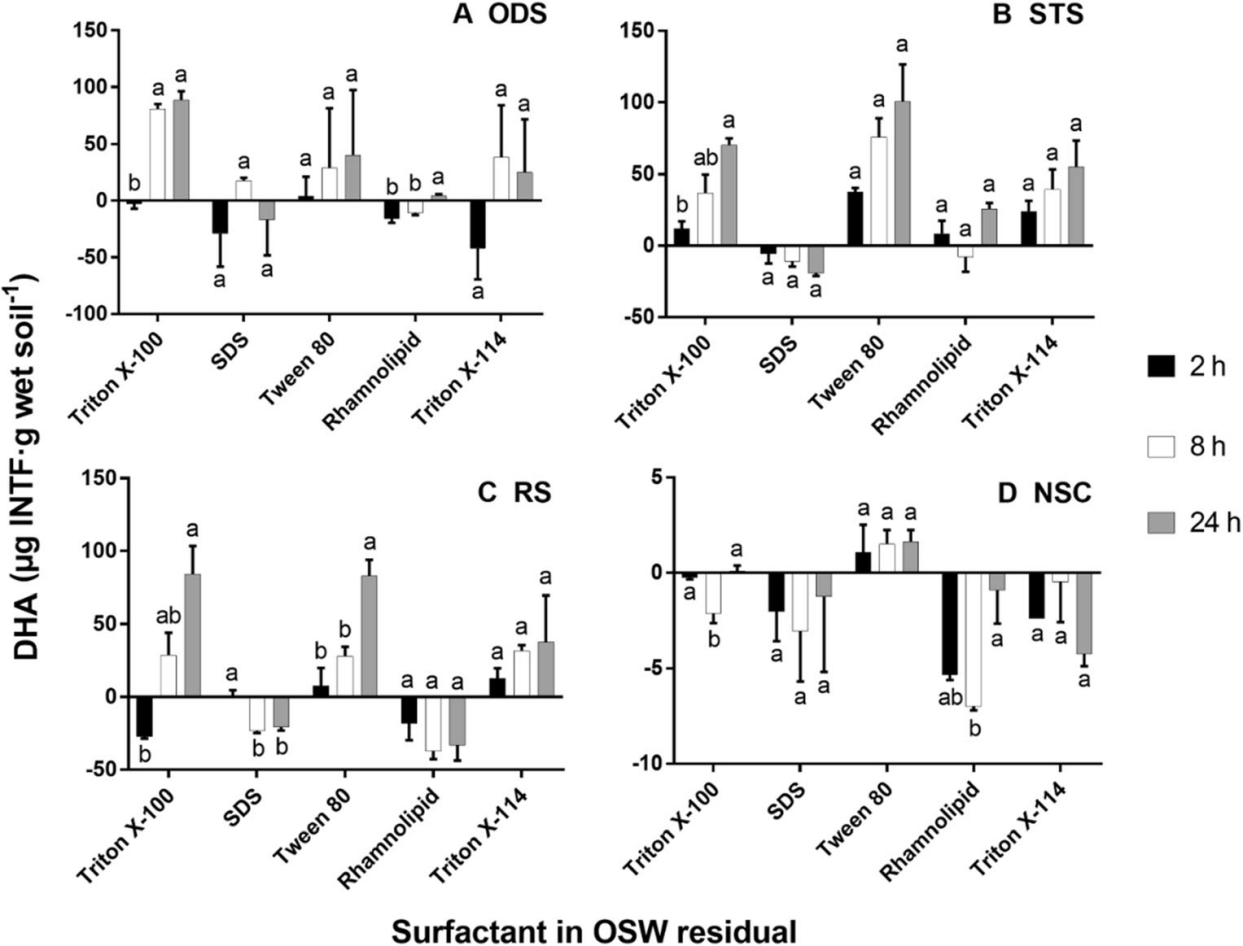
Dehydrogenase activity (DHA) in all oil sludge washing (OSW) residual combinations from ODS, oil drilling sludge (**a**); STS, waste engine oil sludge with a metal removal pre-treatment by gravitational settling (**b**); RS, waste engine oil sludge with a metal removal pre-treatment by centrifugation (**c**); and NSC, oil refinery sludge (**d**) at different incubation times. A Tukey’s test compared the surfactants with incubation times per oil sludge. Values with the same letters are not significantly different (*p* > 0.05). The bars indicate the standard error of the mean, SEM (*n* = 3). INTF: iodonitrotetrazolium formazan. SDS: sodium dodecyl sulphate

The DHA values in all OSW residual combinations are shown in Fig. 2.

The interaction between the factors of OSW residuals and the incubation time was significant (*p* = 0.028). Some OSW residuals (e.g. ODS-SDS in Fig. 2a and RS-SDS in Fig. 2c) had low DHA values at 24 h of incubation which could be due to some early consumption of the INTF produced. Overall, the DHA of the OSW residuals with Triton X-100, Triton X-114 and Tween 80 increased through the incubation time. The values from the NSC residuals with Tween 80 were the highest in the residuals from this sludge (1 (± 2) at 2 h, and 2 (± 1) μg INTF·g wet soil^−1^ at 24 h). Nevertheless, these values were not significantly different from zero (*p* = 0.266 and *p* = 0.058, respectively), which indicated no detectable DHA in all NSC residuals. Also, the DHA in NSC residuals with the other surfactants could not be detected because of negative values. Furthermore, the incubation times in all NSC residuals (Fig. 2d) were not significantly different (*p* = 0.425) which supported the fact that DHA was not detected. The data from the OSW residuals did not imply negative INTF concentration values; instead, these values were obtained because the corrected absorbance was negative after subtracting the sample absorbance from the abiotic controls which had high absorbances. Therefore, other compounds (probably present in this surfactant) could be affecting the detection of the absorbances giving these negative values (Fig. 2). Also, OSW residuals with rhamnolipid had negative values, which was not expected since high DHA values were predicted due to the low toxicity of this biosurfactant (Sekhon Randhawa and Rahman 2014; Irorere et al. 2017). Therefore, the chemical structure of the rhamnolipid could be interfering with the test.

**Fig. 3:**
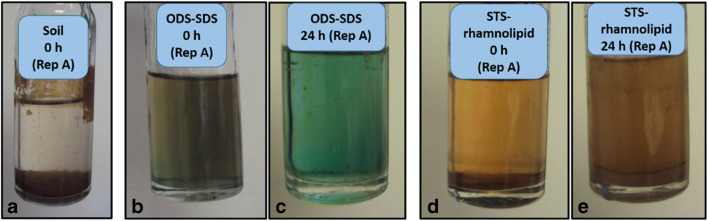
Colouration in the oil sludge washing (OSW) residuals after adding INT to the soil at 0 h (**a**), ODS-SDS at 0 h (**b**) and 24 h (**c**) and STS-rhamnolipid at 0 h (**d**) and 24 h (**e**). ODS: oil drilling sludge. SDS: sodium dodecyl sulphate. INT: iodonitrotetrazolium chloride

Figure 3 shows the colouration in some OSW residual samples during the DHA test at 0 (immediately after adding the INT) and 24 h.

Some of the pictures in Fig. 3 showed samples with negative INTF concentrations values that could be related to chemical interferences unrelated to the DHA test. For instance, ODS (Fig. 3b) and STS (Fig. 3d) residuals had a higher absorbance than soil at 0 h (Fig. 3a). However, the colouration of ODS samples turned to green at 24 h (Fig. 3c) which was detected at 490 nm (Clayden et al. 2012). The absorbance of the DHA test was measured at 464 nm. In this case, some red colouring may have overlapped with the green colour projected from the sample, so the red colour was detected at this wavelength. The fact that tetrazolium salts from the INT can extract copper salts (Altman 1976; Obbard 2001) could be the source of this green colouration found in the ODS residuals. These authors stated that copper ions (Cu^2+^) and formazans in the INTF can form chelates. In fact, some copper trace concentrations were detected in ODS, STS and RS; 8 (± 0.08), 7 (± 0.20) and 12 (± 0.80) μg g^−1^, respectively (Table S2). Some studies that analysed heavy metal-contaminated soils amended with sewage sludges have also reported interference of copper ions in the DHA test (Chander and Brookes 1991; Chander et al. 1995). They used the 2, 3,5-triphenyltetrazolium chloride (TTC), an analogous tetrazolium compound. Additionally, Obbard (2001) reported interference of copper ions using INT in a bench-scale test.

These results established detectable INTF concentrations at 24 h, so this incubation time was chosen to test the DHA of OSW residuals in soil. In addition, STS and Triton X-100 were selected for this next test in soil because their INTF concentrations were easily detected with fewer chemical interactions with the DHA test and no negative values (Figs. 1 and 2).

Also, SDS was analysed due to its toxic effect to microorganisms and aquatic organisms (Martínez-Jerónimo and Muñoz-Mejía 2007; Yilmaz and Icgen 2014), and rhamnolipid was tested at high concentrations (10, 25, and 50%) for comparison due to its low toxicity.

**Fig. 4:**
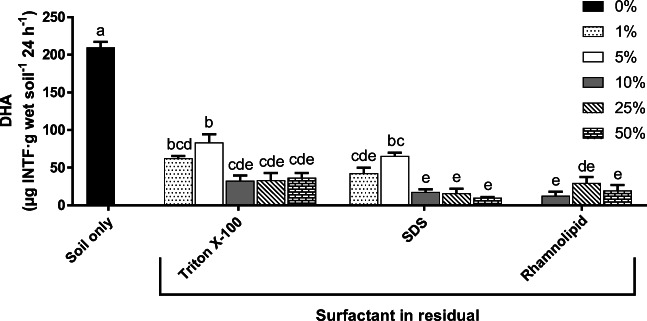
Dehydrogenase activity (DHA) in oil sludge washing (OSW) residuals from the washing of STS (waste engine oil sludge with a metal removal pre-treatment by gravitational settling) in the soil at different percentages. A Tukey’s test compared surfactants with the percentage of OSW residuals. Values with the same letters are not significantly different (*p* > 0.05). The bars indicate the standard error of the mean, SEM (*n* = 3). SDS: sodium dodecyl sulphate. INTF: iodonitrotetrazolium formazan

The total EPH concentrations of the soil amended with the OSW residuals used in this study were in the range of 13 to 21 ppm, and these were not significantly different from the uncontaminated soil and blank reference (sand) which were 13 ppm, except for RS–Triton X-100 (5%), STS–SDS (5 and 10%) and STS–Triton X-100 (10 and 50%). Figure 4 shows the INTF concentrations in the soil of different concentrations of OSW residuals from the washing of STS with Triton X-100, SDS and rhamnolipid.

In general, there was no interference in the DHA tests of the soils with the residuals because the controls did not detect any chemical interactions unrelated to this test. The DHA in the soil sample (0% OSW residual) was significantly higher (200 ± 10) than the OSW residuals (< 110 μg INTF·g wet soil^−1^), so this indicated an apparent impact of the OSW residuals in the soil microbiota. Overall, the INTF production was significantly higher at 1% and 5% (*p* < 0.01) than 10, 25 and 50% OSW residuals (Fig. 4). There was a highly significant effect on the DHA of the type of surfactant and the OSW concentration factors (*p* < 0.01), but their interaction was not significant (*p* = 0.929). In general, the INTF concentrations in the OSW residuals in soil were not significantly different as shown in the 10%, 25% and 50% OSW residual treatments (Fig. 4). In fact, some studies have reported that the concentration of the contaminant in a matrix is not directly proportional to the levels of toxicity in organisms (Domene et al. 2008a, b; Roig et al. 2012; Alvarenga et al. 2016). Therefore, the degree of the stabilization of the contaminant in the matrix could decrease the bioavailability of the contaminant in the oil sludge (Roig et al. 2012; Alvarenga et al. 2016). However, there was not enough evidence to elucidate if the contaminant stabilization in the sludge could be related to low toxicity in this study because the oil sludges were blended with soil for 60 min and left overnight. Therefore, it is suggested to assess the chemical stabilization or weathering (i.e. ageing) effects of the residuals in the soil in future studies. Also, the total EPH concentrations in OSW residual-treated soils were not significantly different to the control soil which suggested that other factors such as heavy metals or the chemical structures of the surfactants were contributing with the toxicity of these residuals.

Surprisingly, the DHA values of rhamnolipid-OSW residuals in soil were significantly lower than the Triton X-100 residuals at 10, 25 and 50%. Even though rhamnolipids had lower toxicity than synthetic surfactants (Irorere et al. 2017), there are some reports on the toxicity of this biosurfactant at high concentrations. For instance, Marecik et al. (2012) reported that high concentrations of rhamnolipid can be toxic by affecting the microbial activity and the germination and growth of plants (e.g. sorghum, mustard and alfalfa). These authors mentioned that the rhamnolipid can alter the permeability of the cell membranes which allows the interaction of the contaminants with the cells (Marecik et al. 2012). Consequently, the interaction between the rhamnolipid and the oil droplets can increase the bioavailability of the contaminant (Mueller et al. 1989; Chrzanowski et al. 2009). Even though there was some toxicity from the rhamnolipid, this biosurfactant is preferred instead of the synthetic surfactants because of its high surface activity and low toxicity and CMC (Liu et al. 2018b).

**Fig. 5:**
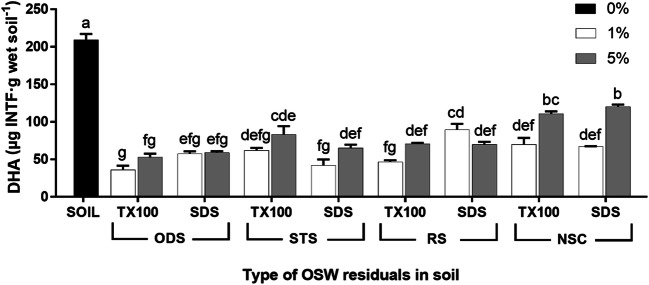
Dehydrogenase activity (DHA) of oil sludge washing (OSW) residuals with Triton X-100 (TX-100) or sodium dodecyl sulphate (SDS) in all sludges amended at different percentages to the soil. A Tukey’s test compared each residual (sludge + surfactant) with the percentage of OSW residuals. Values with the same letters are not significantly different (*p* > 0.05). The bars indicate the standard error of the mean, SEM (*n* = 3). ODS: oil drilling sludge. STS: waste engine oil sludge with a metal removal pre-treatment by gravitational settling. RS: waste engine oil sludge with a metal removal pre-treatment by centrifugation. NSC: oil refinery sludge. INTF: iodonitrotetrazolium formazan

The INTF concentrations in some of the OSW residual combinations (0, 1 and 5%) from the washing of all sludges amended to the soil are shown in Fig. 5.

There were highly significant effects of the OSW residual type and soil concentration factors (*p* < 0.01). The INTF concentration value of the soil was significantly higher than all the INTF concentrations of the OSW residuals (*p* < 0.01), and the highest INTF concentration in the OSW residuals was obtained from NSC-SDS at 5% OSW with 120 (± 5) μg INTF·g wet soil^−1^. This high concentration was probably due to the oil-degrader microorganisms found in the sample as this sample had the highest total EPH concentration as mentioned before. In contrast, the ODS-Triton X-100 residuals at 1% (36 ± 10) and 5% (53 ± 8 μg INTF·g wet soil^−1^) had the lowest concentration values, and these values were significantly different (*p* < 0.01) to the highest INTF concentration mentioned before (Fig. 5). Also, the INTF concentrations at 1% OSW in soil from STS-SDS (42 ± 13 μg INTF·g wet soil^−1^) and RS-Triton X-100 (47 ± 3 μg INTF·g wet soil^−1^) were significantly lower than the concentrations of the NSC-SDS residual (*p* < 0.01). These reductions of DHA in the soil treated with OSW residuals suggested some toxicity to the soil microbiota. Nevertheless, some DHA was found which implies that these residuals can be further treated with bioremediation processes such as landfarming.

### Toxicity of OSW residuals in the germination of ryegrass

Seed germination tests are known to be short-term evaluations of the toxicity of any contaminated matrix, and these tests can mainly assess severe toxicity effects of the contaminant (Banks and Schultz 2005). Due to the considerable range of sludges and surfactants used in the present study, the choices of these factors for the germination test were based on the results obtained in the DHA test. STS and Triton X-100 were chosen because both had the least interference with the DHA test. In addition, ODS was selected for comparison due to the apparent influence of this sludge in the DHA test. Only the 1, 5 and 10% treatments were analysed because concentrations higher than 10% of the liquid OSW residual could saturate and flood the soil exceeding the water holding capacity which could prevent germination. The ranges of the greenhouse temperature and relative humidity throughout the experiment were 15 to 25 °C and 30 to 60%, respectively. The pH values of the soil before and after the germination test in the samples and controls were close to pH neutrality, so there was no toxic effect attributed to it. The germination ended on the seventh day as reported before (Alvarenga et al. 2016). Table 2 shows the ryegrass germination rates in the soil samples amended with OSW residuals.

**Table 2:** Germination rates for different concentrations of oil sludge washing (OSW) residuals (1, 5 and 10%) in soil

OSW residuals	OSW (%)	Germination rate (%)^a^	*p* values^b^ (*H*_1_: μ_d_ < 0)
ODS-Triton X-100	1	76 (± 14)	0.097
ODS-Triton X-100	5	93 (± 2)	0.789
ODS-Triton X-100	10	80 (± 4)	0.018
STS-Triton X-100	1	84 (± 8)	0.113
STS-Triton X-100	5	73 (± 6)	0.017
STS-Triton X-100	10	84 (± 0.01)	0.001

*ODS* oil drilling sludge, *STS* waste engine oil sludge with a metal removal pre-treatment by gravitational settling

^a^All germination rates are mean values with the standard deviation (*n* = 3)

^b^The *p* values from the paired *t* test were obtained by comparing each of the germination rate mean values with the control (92%). The alternative hypothesis (*H*_1_) checked the mean difference (*μ*_d_) between paired OSW treatments (OSW % and surfactant) on each sludge

In general, the germination rates were higher than 70% at all the concentrations. Also, Wei et al. (2019) reported germination rates of ryegrass, tall fescue (*Festuca ovina*) and caragana (*Caragana korshinskii*) higher than 70% at 0.5, 1, 2 and 4% (*w*/*w*) of crude oil in the soil. Moreover, they showed that ryegrass, tall fescue and wheatgrass (*Agropyron cristatum*) can enhance the production of oxidoreductases such as dehydrogenase in the rhizosphere increasing the TPH degradation rates (Wei et al. 2019). The soil (i.e. 0% OSW) and the positive control (reference soil) had germination rates of 92 and 83% (± 9), respectively. There was no sign of phytotoxicity in both controls, and their germination rates were higher than the reported germination rate standard for ryegrass, 75% (USEPA 2006). The negative controls had no germination.

There were no significant differences between the soil samples treated with the residuals from the washing of the ODS and STS sludges in all concentrations (*p* = 0.764). Besides, the germination rate of the 5% Triton X-100 control (83% ± 10) was not significantly different to the rate of the 5% OSW residuals from both sludges (ODS: *p* = 0.157; STS: *p* = 0.073). However, an apparent highly significant negative effect of the residuals in the germination rates of the STS-Triton X-100 (5%), ODS-Triton X-100 (10%) and STS-Triton X-100 (10%) residuals in soil was detected (*p* < 0.01). Therefore, this negative effect in the germination of the ryegrass at high concentrations implies that low concentrations (e.g. 1%) should be considered for phytoremediation purposes. As mentioned before in the DHA test, this negative effect cannot be due to the EPH contamination because it was less than 20 ppm. Thus, the co-contamination with heavy metals and the surfactant solution could be influencing the toxic effect of these residuals. Future studies can evaluate the individual effect of heavy metals and surfactant solutions to establish which compound is the most toxic.

## Conclusions

This study analysed the toxicity of the OSW residuals in soil microbiota and ryegrass germination, and it showed the relevance of using ecotoxicity assessments (to assess the impact of the bioavailable contaminant into the organisms) combined with chemical tests. To our knowledge, there have been no studies that analysed specifically the toxicity in these residuals from different sludges in a comprehensive context. In general, the toxic effects of the residuals were not high in the soil microbiota and ryegrass germination. Therefore, the dehydrogenase activity of the soil microbiota showed a 40% decrease in average and the ryegrass germination rate was higher than 70%.

The DHA test was successful when assessing the OSW residual-amended soils. Nevertheless, when the oil sludges and the OSW residuals were measured directly, there was a high interference with the DHA test (e.g. copper traces in the OSW residuals) which showed the contaminant-related complexity of the oil sludge matrix. The copper and other heavy metals could be washed out in the residuals after the OSW process contributing not only with the interference with the DHA test but also with the toxic effect of the residuals. Therefore, the DHA test should be done only by mixing the residuals with a soil matrix, which indeed is pertinent because it simulates a bioremediation scenario. For this test, STS and Triton X-100 were used because their DHA values were easily detected with no apparent interference at all. SDS and rhamnolipid were used due to their reported high and low toxicity, respectively. Surprisingly, the INTF values of the 10, 25, and 50% rhamnolipid-OSW residuals were significantly lower than the Triton X-100 residuals, which showed the toxicity of this biosurfactant at high concentrations. Nevertheless, rhamnolipids are preferred in oil sludge washing processes due to its high surface activity and low toxicity compared with other synthetic surfactants.

To conclude, the toxicity assessments (DHA and germination of ryegrass) showed that the OSW residuals can follow further treatment via bioremediation techniques such as landfarming and phytoremediation. Therefore, both toxicity tests can be used as fast and simple assessments to determine if any residual from the washing is suitable for a final biotreatment.

## Supplementary Information

ESM 1 (DOCX 36.2 kb)

## Data Availability

The datasets generated and/or analysed during the current study are available in the Mendeley data repository (10.17632/y4kgjrp6db.1).

## References

[CR1] Altman FP (1976). Tetrazolium salts and formazans. Prog Histochem Cytochem.

[CR2] Alvarenga P, Palma P, Gonçalves AP, Fernandes RM, Cunha-Queda AC, Duarte E, Vallini G (2007). Evaluation of chemical and ecotoxicological characteristics of biodegradable organic residues for application to agricultural land. Environ Int.

[CR3] Alvarenga P, Mourinha C, Farto M, Palma P, Sengo J, Morais M-C, Cunha-Queda C (2016). Ecotoxicological assessment of the potential impact on soil porewater, surface and groundwater from the use of organic wastes as soil amendments. Ecotoxicol Environ Saf.

[CR4] ASTM-E1963-09 (2014). Standard guide for conducting terrestrial plant toxicity tests.

[CR5] Banks MK, Schultz KE (2005). Comparison of plants for germination toxicity tests in petroleum-contaminated soils. Water Air Soil Pollut.

[CR6] Barrutia O, Garbisu C, Epelde L, Sampedro MC, Goicolea MA, Becerril JM (2011). Plant tolerance to diesel minimizes its impact on soil microbial characteristics during rhizoremediation of diesel-contaminated soils. Sci Total Environ.

[CR7] Besalatpour A, Khoshgoftarmanesh AH, Hajabbasi MA, Afyuni M (2008). Germination and growth of selected plants in a petroleum contaminated calcareous soil. Soil Sediment Contam Int J.

[CR8] Campos JA, Peco JD, García-Noguero E (2019). Antigerminative comparison between naturally occurring naphthoquinones and commercial pesticides. Soil dehydrogenase activity used as bioindicator to test soil toxicity. Sci Total Environ.

[CR9] Chander K, Brookes PC (1991). Is the dehydrogenase assay invalid as a method to estimate microbial activity in copper-contaminated soils?. Soil Biol Biochem.

[CR10] Chander K, Brookes PC, Harding SA (1995). Microbial biomass dynamics following addition of metal-enriched sewage sludges to a sandy loam. Soil Biol Biochem.

[CR11] Chrzanowski Ł, Wick LY, Meulenkamp R, Kaestner M, Heipieper HJ (2009). Rhamnolipid biosurfactants decrease the toxicity of chlorinated phenols to Pseudomonas putida DOT-T1E. Lett Appl Microbiol.

[CR12] Clayden J, Greeves N, Warren S (2012). Organic chemistry.

[CR13] Cook RL, Hesterberg D (2013). Comparison of trees and grasses for rhizoremediation of petroleum hydrocarbons. International Journal of Phytoremediation.

[CR14] Domene X, Alcañiz JM, Andrés P (2008). Comparison of solid-phase and eluate assays to gauge the ecotoxicological risk of organic wastes on soil organisms. Environ Pollut.

[CR15] Domene X, Ramírez W, Mattana S, Alcañiz JM, Andrés P (2008). Ecological risk assessment of organic waste amendments using the species sensitivity distribution from a soil organisms test battery. Environ Pollut.

[CR16] Duan M, Wang X, Fang S, Zhao B, Li C, Xiong Y (2018). Treatment of Daqing oily sludge by thermochemical cleaning method. Colloids Surf A Physicochem Eng Asp.

[CR17] European Parliament, E (2008). Directive 2008/98/EC of the European Parliament and of the council on waste and repealing certain directives. Official Journal of the European Union, L.

[CR18] Greene JC, Bartels CL, Warren-Hicks WJ, Parkhurst BR, Linder GL, Peterson SA, Miller WE (1989). EPA 600/3–88/029: protocols for short term toxicity screening of hazardous waste sites.

[CR19] Gumerov FM, Khairutdinov VF, Akhmetzyanov TR, Gabitov FR, Zaripov ZI, Farakhov MI, Mukhutdinov AV (2017). Supercritical fluid propane-butane extraction treatment of oil sludge. Russian Journal of Physical Chemistry B.

[CR20] Hu G, Li J, Zeng G (2013). Recent development in the treatment of oily sludge from petroleum industry – a review. J Hazard Mater.

[CR21] Huang H, Tang J, Niu Z, Giesy JP (2019). Interactions between electrokinetics and rhizoremediation on the remediation of crude oil-contaminated soil. Chemosphere.

[CR22] Hwa SC, Shang LY, Wasan S, Loong CY (2016) *A method of treating oily solid particles. U.S. Patent Application No. US 2016/0319200 A1*. Available at: https://patents.google.com/patent/US20160319200A1/en (Accessed: August 2nd 2018)

[CR23] Irorere VU, Tripathi L, Marchant R, McClean S, Banat IM (2017). Microbial rhamnolipid production: a critical re-evaluation of published data and suggested future publication criteria. Appl Microbiol Biotechnol.

[CR24] Jasmine J, Mukherji S (2015). Characterization of oily sludge from a refinery and biodegradability assessment using various hydrocarbon degrading strains and reconstituted consortia. J Environ Manag.

[CR25] Jin Y, Zheng X, Chi Y, Ni M (2013). Rapid, accurate measurement of the oil and water contents of oil sludge using low-field NMR. Ind Eng Chem Res.

[CR26] Kaczyńska G, Borowik A, Wyszkowska J (2015). Soil dehydrogenases as an indicator of contamination of the environment with petroleum products. Water Air Soil Pollut.

[CR27] Kaimi E, Mukaidani T, Miyoshi S, Tamaki M (2006). Ryegrass enhancement of biodegradation in diesel-contaminated soil. Environ Exp Bot.

[CR28] Kirk JL, Klironomos JN, Lee H, Trevors JT (2005). The effects of perennial ryegrass and alfalfa on microbial abundance and diversity in petroleum contaminated soil. Environ Pollut.

[CR29] Kriipsalu M, Marques M, Maastik A (2008). Characterization of oily sludge from a wastewater treatment plant flocculation-flotation unit in a petroleum refinery and its treatment implications. Journal of Material Cycles and Waste Management.

[CR30] Kuppusamy S, Thavamani P, Venkateswarlu K, Lee YB, Naidu R, Megharaj M (2017). Remediation approaches for polycyclic aromatic hydrocarbons (PAHs) contaminated soils: technological constraints, emerging trends and future directions. Chemosphere.

[CR31] Liang J, Zhao L, Hou W (2017). Solid effect in chemical cleaning treatment of oily sludge. Colloids Surf A Physicochem Eng Asp.

[CR32] Lin D, Xing B (2007). Phytotoxicity of nanoparticles: inhibition of seed germination and root growth. Environ Pollut.

[CR33] Liu C, Zhang Y, Sun S, Huang L, Yu L, Liu X, Lai R, Luo Y, Zhang Z, Zhang Z (2018). Oil recovery from tank bottom sludge using rhamnolipids. J Pet Sci Eng.

[CR34] Liu G, Zhong H, Yang X, Liu Y, Shao B, Liu Z (2018). Advances in applications of rhamnolipids biosurfactant in environmental remediation: a review. Biotechnol Bioeng.

[CR35] MAFF (1986) *The Analysis of Agricultural Materials. Reference Book 427.* Third Edition edn. London, UK: Ministry of Agriculture, Fisheries and Food (MAFF). Her Majesty's Stationery Office (HMSO)

[CR36] Małachowska-Jutsz A, Matyja K (2019). Discussion on methods of soil dehydrogenase determination. Int J Environ Sci Technol.

[CR37] Mansour H, Saber M, Awad F, Zaghloul A (2019). Dehydrogenase activity and zinc equivalent parameters as indicators for potential toxic elements remediation in polluted soil ecosystem. Bioremediation Journal.

[CR38] Marecik R, Wojtera-Kwiczor J, Ławniczak Ł, Cyplik P, Szulc A, Piotrowska-Cyplik A, Chrzanowski Ł (2012). Rhamnolipids increase the phytotoxicity of diesel oil towards four common plant species in a terrestrial environment. Water Air Soil Pollut.

[CR39] Martínez-Jerónimo F, Muñoz-Mejía G (2007). Evaluation of the sensitivity of three cladoceran species widely distributed in Mexico to three reference toxicants, *Journal of Environmental Science and Health*. Part A.

[CR40] Mazen A, Faheed FA, Ahmed AF (2010). Study of potential impacts of using sewage sludge in the amendment of desert reclaimed soil on wheat and jews mallow plants. Braz Arch Biol Technol.

[CR41] Mirghaffari N (2017) Treatment and recycling of oily sludges produced in the petroleum industry. *2017 International Conference on Environmental Impacts of the Oil and Gas Industries: Kurdistan Region of Iraq as a Case Study (EIOGI)*, 17–19 April 2017, 1–2

[CR42] Mueller JG, Chapman PJ, Pritchard PH (1989). Creosote-contaminated sites. Their potential for bioremediation. Environmental Science & Technology.

[CR43] Muratova AY, Dmitrieva TV, Panchenko LV, Turkovskaya OV (2008). Phytoremediation of oil-sludge–contaminated soil. International Journal of Phytoremediation.

[CR44] Nezhdbahadori F, Abdoli MA, Baghdadi M, Ghazban F (2018). A comparative study on the efficiency of polar and non-polar solvents in oil sludge recovery using solvent extraction. Environ Monit Assess.

[CR45] Obbard PJ (2001). Measurement of dehydrogenase activity using 2-p-iodophenyl-3-p-nitrophenyl-5-phenyltetrazolium chloride (INT) in the presence of copper. Biol Fertil Soils.

[CR46] Olson PE, Reardon KF, Pilon-Smits EAH, McCutcheon SC, Schnoor JL (2003). Ecology of rhizosphere bioremediation. Phytoremediation: transformation and control of contaminants Enviromental science and technology: a Wiley-Interscience series of texts and monographs.

[CR47] Onwosi CO, Odimba JN, Igbokwe VC, Nduka FO, Nwagu TN, Aneke CJ, Eke IE (2019) Principal component analysis reveals microbial biomass carbon as an effective bioindicator of health status of petroleum-polluted agricultural soil, *Environmental Technology*, pp. 1–13 10.1080/09593330.2019.1603252 30982397

[CR48] Ramirez D, Collins CD (2018). Maximisation of oil recovery from an oil-water separator sludge: influence of type, concentration, and application ratio of surfactants. Waste Manag.

[CR49] Ramirez D, Kowalczyk RM, Collins CD (2019). Characterisation of oil sludges from different sources before treatment: high-field nuclear magnetic resonance (NMR) in the determination of oil and water content. J Pet Sci Eng.

[CR50] Ramirez D, Shaw LJ, Collins CD (2020) Oil sludge washing with surfactants and co-solvents: oil recovery from different types of oil sludges. Environ Sci Pollut Res. 10.1007/s11356-020-10591-9 10.1007/s11356-020-10591-9 PMC7838146 32974830

[CR51] Roig N, Sierra J, Nadal M, Martí E, Navalón-Madrigal P, Schuhmacher M, Domingo JL (2012). Relationship between pollutant content and ecotoxicity of sewage sludges from Spanish wastewater treatment plants. Sci Total Environ.

[CR52] Rosen, M. J. and Kunjappu, J. T. (2012) *Surfactants and Interfacial Phenomena.* Hoboken, NJ, USA: Wiley: a John Wiley and Sons, Inc., publication

[CR53] Sakai S-I, Yoshida H, Hirai Y, Asari M, Takigami H, Takahashi S, Tomoda K, Peeler MV, Wejchert J, Schmid-Unterseh T, Douvan AR, Hathaway R, Hylander LD, Fischer C, Oh GJ, Jinhui L, Chi NK (2011). International comparative study of 3R and waste management policy developments. Journal of Material Cycles and Waste Management.

[CR54] Sekhon Randhawa KK, Rahman PKSM (2014). Rhamnolipid biosurfactants—past, present, and future scenario of global market. Front Microbiol.

[CR55] Serrano A, Gallego M, González JL, Tejada M (2008). Natural attenuation of diesel aliphatic hydrocarbons in contaminated agricultural soil. Environ Pollut.

[CR56] Shaw LJ and Burns RG (2006) Enzyme activity profiles and soil quality, in Bloem, J., Hopkins, D. and Benedetti, A. (eds.) *Microbiological Methods for Assessing Soil Quality*. Wallingford, UK: CABI (Centre for Agriculture and Biosciences International), pp. 158–172

[CR57] Singh RP, Agrawal M (2007). Effects of sewage sludge amendment on heavy metal accumulation and consequent responses of Beta vulgaris plants. Chemosphere.

[CR58] Suleimanov RR, Gabbasova IM, Sitdikov RN (2005). Changes in the properties of oily gray forest soil during biological reclamation. Biol Bull.

[CR59] Tang J, Wang R, Niu X, Zhou Q (2010). Enhancement of soil petroleum remediation by using a combination of ryegrass (Lolium perenne) and different microorganisms. Soil Tillage Res.

[CR60] USEPA 2006. Ecological Effects Test. Guidelines. OPPTS 850.4225 Seedling Emergence Tier II. EPA 712-C-96-363. *US Environmental Protection Agency.* Washington, DC, USA

[CR61] Wang S, Zhang C, Lu G, Li F, Guo G (2016). Screening of herbaceous plants for peat-enhanced rehabilitation of contaminated soil with oily sludge. International Journal of Phytoremediation.

[CR62] Wang B, Xie H-L, Ren H-Y, Li X, Chen L, Wu B-C (2019). Application of AHP, TOPSIS, and TFNs to plant selection for phytoremediation of petroleum-contaminated soils in shale gas and oil fields. J Clean Prod.

[CR63] Wei Y, Wang Y, Duan M, Han J, Li G (2019). Growth tolerance and remediation potential of six plants in oil-polluted soil. J Soils Sediments.

[CR64] Wesson LL, Harwell JH (2000) Surfactant adsorption in porous media, in Schramm, L.L. (ed.) *Surfactants: fundamentals and applications in the petroleum industry.* First paperback edition ed. New York, USA: Cambridge University Press, pp. 121–158

[CR65] Wilke BM, Riepert F, Koch C, Kühne T (2008). Ecotoxicological characterization of hazardous wastes. Ecotoxicol Environ Saf.

[CR66] Wolf DC, Cryder Z, Khoury R, Carlan C, Gan J (2020) Bioremediation of PAH-contaminated shooting range soil using integrated approaches, Science of The Total Environment, pp. 138440 10.1016/j.scitotenv.2020.138440 32315846

[CR67] Yilmaz F, Icgen B (2014). Characterization of SDS-degrading Delftia acidovorans and in situ monitoring of its temporal succession in SDS-contaminated surface waters. Environ Sci Pollut Res.

[CR68] Zhang J, Li J, Thring RW, Hu X, Song X (2012). Oil recovery from refinery oily sludge via ultrasound and freeze/thaw. J Hazard Mater.

