# Supplementary material for: Ecotoxicity of oil sludges and residuals from their washing with surfactants: soil dehydrogenase and ryegrass germination tests
Source: Environ Sci Pollut Res Int. 2020 Nov 11;28(11):13312–22. doi: 10.1007/s11356-020-11300-2 (PMC7943489; doi:10.1007/s11356-020-11300-2)
Supplement: Supplementary file 1 — (DOCX 36.2 kb) [file 11356_2020_11300_MOESM1_ESM.docx]

# Ecotoxicity of oil sludges and residuals from their washing with surfactants: Soil dehydrogenase and ryegrass germination tests

Diego Ramirez ^a,^ , Liz J. Shaw ^a^, Chris D. Collins ^a,*^

^a^ Department of Geography and Environmental Science, University of Reading, Reading, RG6 6DW, UK

* Corresponding author.

*E-mail addresses:* [diego.ramirez.guerrero@gmail.com](mailto:diego.ramirez.guerrero@gmail.com) (D. Ramirez), [e.j.shaw@reading.ac.uk](mailto:e.j.shaw@reading.ac.uk) (L. J. Shaw), [c.d.collins@reading.ac.uk](mailto:c.d.collins@reading.ac.uk) (C.D. Collins).

Table S 1. Physicochemical properties of the oil sludges.

| Sludge |  | | Wet and dry contents ^2^ | | | Extractable Petroleum Hydrocarbons (EPH) ^3^ | | | |
| --- | --- | --- | --- | --- | --- | --- | --- | --- | --- |
|  | **Oil ^1^**  **(%)** | **Water ^1^**  **(%)** | **Wet** | **Solids (dry)** | **Organic (dry)** | **C_10_-C_18_**  **aliphatic (%)** | **C_19_-C_36_**  **aliphatic (%)** | **C_11_-C_22_ aromatic (%)** | **Total EPH**  **(ppm)** |
| ODS | 1 (±0.26) | 99 (±0.26) | 13 (±0.02) | 86 (±0.11) | 1 (±0.10) | 98 | 0.53 | 1.47 | 6,000 (± 145) |
| STS | 50 (±14) | 50 (±14) | 41 (±0.16) | 35 (±0.16) | 24 (±0.09) | 13 | 83 | 4 | 1,550 (± 506) |
| RS | 39 (±1) | 61 (±1) | 35 (±2) | 38 (±0.17) | 27 (±2) | 10 | 85 | 5 | 949 (± 392) |
| NSC | 88 (± 11) | 12 (± 11) | 60 (±2) | 1 (±0.07) | 39 (±2) | 69 | 30 | 1 | 68,000 (± 6,070) |

**^1^** Calculated by high-field nuclear magnetic resonance (NMR).

**^2^** Calculated by the oven-drying method.

**^3^** Determined by gas chromatography-flame ionisation detector (GC-FID).

The mean (*n* = 3) with the standard deviation is shown in parentheses.

The analyses of the oil sludges were performed in a previous study (Ramirez, Kowalczyk and Collins, 2019).

Table S 2. Trace metal elements concentrations (µg·g^-1^ dry matter or ppm) of the oil sludges analysed.

|  | Sludge | | |
| --- | --- | --- | --- |
| Element | **ODS** | **STS** | **RS** |
| Al | 506 (±20) | 104 (±3) | 116 (±1) |
| *As^*^* | 1 (±0.11) | 0.10 (±0.03) | 0.13 (±0.09) |
| Ba | 85 (±13) | 40 (±2) | 41 (±5) |
| Ca | 4,369 (±2505) | 5,260 (±3033) | 11,093 (±160) |
| *Cd^*^* | 0.08 (±0.01) | 0.02 (±0.01) | 0.02 (±0.01) |
| *Co^*^* | 0.27 (±0.02) | 0.66 (±0.01) | 0.21 (±0.01) |
| *Cr^*^* | 2 (±0.19) | 9 (±0.30) | 9 (±0.07) |
| *Cu^*^* | 8 (±0.08) | 7 (±0.20) | 12 (±0.80) |
| Fe | 1,536 (±867) | 16,146 (±252) | 13,915 (±278) |
| K | 85 (±3) | 81 (±5) | 116 (±6) |
| Li | 0.80 (±0.01) | 0.65 (±0.05) | 0.86 (±0.03) |
| Mg | 220 (±10) | 235 (±3) | 276 (±9) |
| Mn | 179 (±3) | 23 (±0.47) | 21 (±0.41) |
| Na | 65 (±17) | 168 (±16) | 241 (±3) |
| *Ni^*^* | 2 (±0.23) | 2 (±0.14) | 2 (±0.35) |
| *Pb^*^* | 45 (±2) | 9 (±0.93) | 9 (±2) |
| Sr | 93 (±6) | 6 (±0.24) | 7 (±0.07) |
| *Zn^*^* | **1,683 (±22)** | **3,074 (±1814)** | **6,336 (±85)** |

***** These elements are heavy metals (Shaheen *et al.*, 2016).

Values in bold are over the limit of the acceptable standards of landfilling of hazardous waste established by the European Union: As (25 µg·g^-1^), Cd (5), Cr (70), Cu (100), Ni (40), Pb (50), Zn (200) (Kriipsalu, Marques and Maastik, 2008).

These analyses were performed in a previous study (Ramirez, Kowalczyk and Collins, 2019).

**REFERENCES**

Kriipsalu, M., Marques, M. and Maastik, A. (2008) 'Characterization of oily sludge from a wastewater treatment plant flocculation-flotation unit in a petroleum refinery and its treatment implications', *Journal of Material Cycles and Waste Management,* 10(1), pp. 79-86.

Ramirez, D., Kowalczyk, R. M. and Collins, C. D. (2019) 'Characterisation of oil sludges from different sources before treatment: High-field nuclear magnetic resonance (NMR) in the determination of oil and water content', *Journal of Petroleum Science and Engineering,* 174, pp. 729-737.

Shaheen, S. M., Tsadilas, C. D., Ok, Y. S. and Rinklebe, J. (2016) 'Potential Mobility, Bioavailability, and Plant Uptake of Toxic Elements in Temporary Flooded Soils', in Rinklebe, J., Knox, A.S. and Paller, M. (eds.) *Trace Elements in Waterlogged Soils and Sediments*. Boca Raton, FL, USA: CRC Press: Taylor & Francis Group.
